# The effect of moisturizers on acute radiation dermatitis: A systematic review and meta-analysis

**DOI:** 10.1097/MD.0000000000047688

**Published:** 2026-02-20

**Authors:** Wangli Wu, Lin Yao, Saisai Liu, Xiaojing Sun, Xiuwen Zhang, Xuehong Yu

**Affiliations:** a Dermatology, Weihai Municipal Hospital, Cheeloo College of Medicine, Shandong University, Weihai, China.

**Keywords:** acute radk, ermatitis, systematic review

## Abstract

**Background:**

This systematic review and meta-analysis aims to evaluate the effectiveness of moisturizers in preventing acute radiation dermatitis (ARD) in patients undergoing radiotherapy (RT).

**Method:**

We searched the PubMed, Cochrane Library, Embase, and Web of Science databases for literature from inception until August 26, 2025, identifying 13 randomized controlled trials that explored the impact of moisturizers on ARD. Statistical analyses were performed using RevMan 5.3 and STATA 15 software. The Cochrane risk of Bias tool (RoB 2.0) was used to evaluate the risk of bias in the included studies.

**Result:**

The primary outcomes measured included the severity of ARD, and the secondary outcomes were skin-related quality of life, pruritus symptoms, and skin water content. From 1430 records screened, 13 studies were included investigating the effectiveness of moisturizers in patients undergoing RT. A total of 1203 patients with breast cancer or head and neck cancer were involved, including 603 patients in the intervention group and 600 patients in the control group. Results indicated that the probability of not developing ARD was higher in the moisturizer group compared to the control group (RR = 1.73, 95% CI [1.11–2.70], *P* = .02). The incidence of pruritus was lower in the moisturizer group than in the control group (RR = 0.69, 95% CI [0.53–0.90], *P* = .007), with a significantly lower probability of severe pruritus (≥ Grade 2) (RR = 0.06, 95% CI [0.01–0.67], *P* = .02). However, there were no statistically significant differences between the moisturizer and control groups regarding quality of life and water content.

**Conclusion:**

Current evidence suggests that the use of moisturizers may have a positive effect on radiation dermatitis.

## 1. Introduction

Acute radiation dermatitis (ARD) is one of the most frequently observed adverse effects of radiotherapy (RT), affecting a substantial proportion of cancer patients. Studies indicate that up to 95% of patients undergoing RT develop some form of ARD, particularly among those receiving treatment for breast or head and neck cancers.^[1,2]^ ARD not only significantly impairs patients’ quality of life (QOL) but may also lead to treatment interruptions, thereby compromising oncological outcomes.^[1]^

ARD typically manifests within hours to weeks after initiation of RT, presenting as erythema, dry or moist desquamation, and in severe cases, ulceration and necrosis.^[3]^ Its pathogenesis involves DNA damage in rapidly dividing keratinocytes, microvascular injury, and a robust inflammatory response, ultimately impairing skin barrier function.^[4]^

Although several international guidelines recommend various topical agents and dressings for preventing and managing ARD, clinical practice remains heterogeneous due to limited high-quality evidence and inconsistent study results.^[5–7]^ While topical corticosteroids are commonly used, their long-term application is constrained by potential side effects, especially on broken skin.^[8]^ Nonsteroidal agents, including hyaluronic acid, aloe vera, and urea-based emollients, are also employed, yet their efficacy is not firmly established.^[9–12]^

The role of moisturizers, in particular, remains controversial. Some guidelines endorse their use for maintaining skin integrity during RT,^[13,14]^ while others do not recommend them.^[15]^ A previous meta-analysis by Sekiguchi et al suggested a potential benefit of moisturizers in reducing severe ARD, but the conclusion was limited by the inclusion of only 6 randomized trials and a small sample size.^[16]^

Therefore, the present systematic review and meta-analysis aims to synthesize updated evidence from randomized controlled trials (RCTs) to evaluate the efficacy of moisturizers in preventing ARD and improving related symptoms, thereby providing a clearer basis for clinical practice.

## 2. Methods

This study was conducted in accordance with the Cochrane Handbook for Systematic Reviews of Interventions (available at http://training.cochrane.org/handbook) and the preferred reporting items for systematic reviews and meta-analyses (PRISMA) statement.^[17]^ Our study protocol has been registered with PROSPERO, registration number CRD42024523495. Ethical approval or patient consent was not required for this study.

### 2.1. Search strategy

Two researchers (Wu Wangli and Yao Lin) independently searched the PubMed, Cochrane Library, Embase, and Web of Science databases from inception to August 26, 2025. The search strategy combined subject headings and free-text terms, with adjustments made according to the specific requirements of each database. Search terms included “Moisturizer,” “Radiodermatitis,” “Skin Cream,” “Gels,” “Emollients,” “Hyaluronic Acid,” and “Aloe vera gel,” connected using Boolean logic operators “and” and “or” (see Supplementary Material 1, Supplemental Digital Content, https://links.lww.com/MD/R403 for detailed search strategy). Additionally, we reviewed the reference lists of retrieved studies to identify other relevant research.

### 2.2. Inclusion and exclusion criteria

Following the flowchart, trials meeting the following eligibility criteria were included: The study population consisted of all patients who were clinically diagnosed and required radiation therapy, and who met the indications for moisture application; Control groups received either standardized care, including the use of a placebo, or no treatment. The experimental group received standard moisturizer treatment, defined as a product primarily intended for moisturizing rather than possessing significant anti-inflammatory or antioxidant pharmacological effects (such as corticosteroids). Aloe vera is known for its skin-moisturizing and barrier-integrity properties,^[18]^ and is categorized as a moisturizer in some publications.^[19,20]^ Additionally, aloe vera gel is commonly used as a daily moisturizing skincare product in China. Therefore, it was also included as an intervention in the experimental group; At least 1 outcome of interest was reported; Studies must be published in English. Exclusion criteria included: systematic reviews, meta-analyses, case reports, conference papers, and letters; literature inaccessible in full-text, non-original data literature, gray literature, etc.

### 2.3. Literature screening and data extraction

EndnoteX9 software was utilized for literature management. After removing duplicate records, 2 researchers (Wu Wangli and Yao Lin) independently screened titles and abstracts. Subsequently, full texts were obtained to determine the final inclusion of studies. In case of disagreement during literature screening, a consensus was reached through discussion with a third researcher (Sun Xiaojing). Basic information from the selected studies was extracted and cross-checked before being saved in Excel. The extracted information mainly includes the title, first author, publication year, country, sample size, patient age, intervention methods, follow-up period, outcome indicators, and measurement methods. Primary outcome indicators included the occurrence rate and grading of radiodermatitis (ARD grading), primarily assessed using the RTOG grading system to evaluate the effectiveness of radiodermatitis intervention. The RTOG grading system evaluates changes in skin color, pain sensation, skin integrity, etc., and is one of the most widely used standards internationally.^[21]^ If other scales were utilized in the studies, they were reclassified according to the RTOG system. Secondary outcome measures included: Grading of itching symptoms; QOL; skin water content, etc.

### 2.4. Risk of bias

The Cochrane Risk of Bias Assessment Tool, RoB2.0,^[22]^ was employed to evaluate the quality of the included studies. The assessment covered 5 aspects: bias in the randomization process; deviations from intended interventions; bias due to missing outcome data; bias in outcome measurement; bias in the selection of the reported result. These aspects were categorized into 3 levels: low risk, some concerns, and high risk. Two researchers independently assessed the risk of bias and checked for consistency. Discrepancies were resolved through discussion or adjudicated by a third researcher to reach a consensus.

### 2.5. Statistical analysis

STATA 15.0 and RevMan 5.3 were used to perform statistical analyses. A meta-analysis was performed to statistically summarize data when studies were combinable and relatively homogeneous in design, intervention, and outcomes. For dichotomous variables such as the occurrence rate and grading of radiodermatitis and the grading of itching severity, event numbers were entered, and relative risk (RR) was used as the effect size, along with a 95% confidence interval. Continuous data such as QOL and water content were entered in the mean and standard deviation formats. When there were differences in measurement methods or units between groups that might lead to significant numerical differences, standardized mean difference was used as the measurement unit. If measurement methods were consistent between groups, mean difference was used as the effect size. Both were reported with a 95% confidence interval.

When conducting meta-analyses, the fixed-effects model assumes 1 true effect size underlies all the studies in the meta-analysis, while the random-effects model assumes that the true effect could vary from study to study due to the differences (heterogeneity) among studies. And in the random-effects models, smaller studies have relatively greater weight than in fixed-effect models. So we used a random-effect model for all outcomes.^[23]^ Subsequently, subgroup analyses were performed to investigate potential sources of between-study heterogeneity. We planned to assess publication bias by visual inspection of funnel plots and Egger regression test when ten or more studies were included in the meta-analysis.^[24]^ If fewer than ten studies were available, these methods were not performed because of their limited statistical power. Sensitivity analysis was performed using the “leave-one-out” method to determine new effect size, thereby assessing the robustness and stability of the results.

To rate the certainty of evidence for each outcomes, 2 researchers (Wu Wangli and Yao Lin) used the Grading of Recommendations Assessment, Development, and Evaluation (GRADE) criteria. In the system, according to 5 domains: limitations of design, inconsistency, indirectness, imprecision, publication bias, quality of the evidence varies from 4 levels: high quality, moderate quality, low quality, and very low quality.^[25]^

## 3. Results

### 3.1. Literature search results

**Figure 1.**
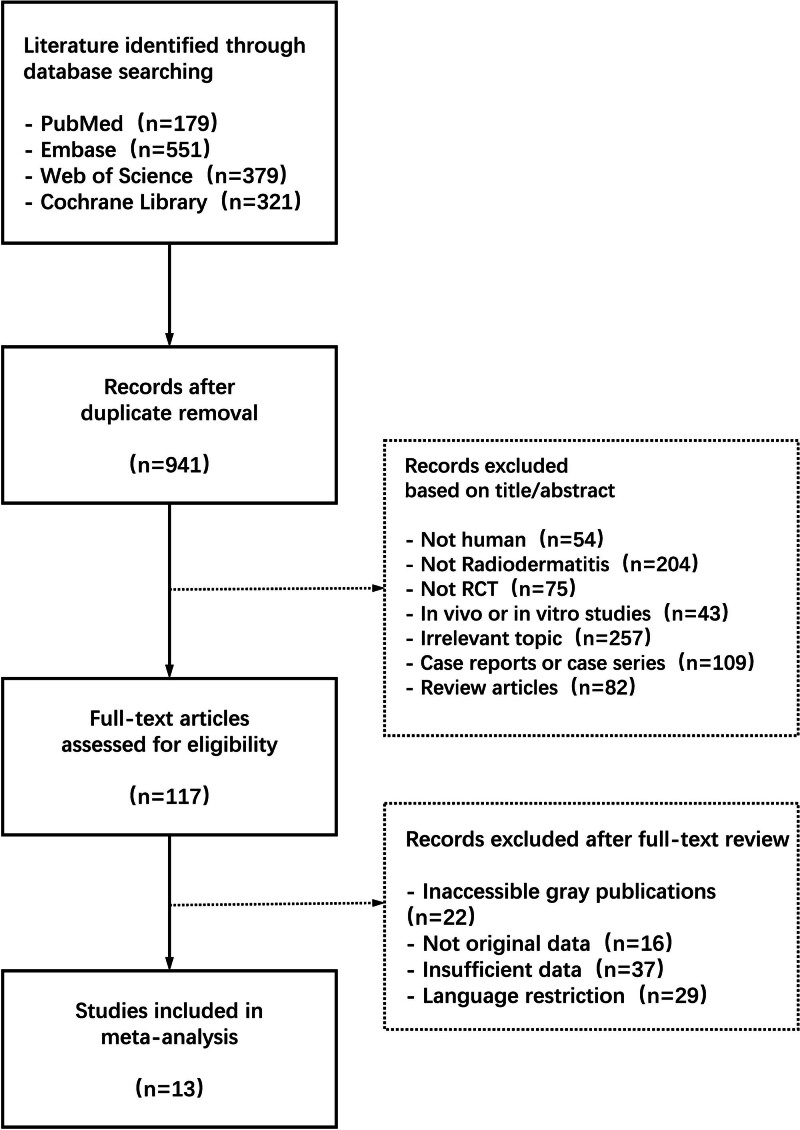
Study flow diagram.

A total of 1430 articles were identified through systematic searches, including 179 from PubMed, 551 from Embase 379 from Web of Science, and 321 from The Cochrane Library. After screening and full-text assessment, 13 articles were included in the study. The flowchart and results of the literature search process are illustrated in Figure 1.

### 3.2. Basic characteristics of included studies

Thirteen studies in total^[26–38]^ were included, all RCTs. These studies were published between 1996 and 2025, with a total sample size of 1203 patients, including 603 patients in the intervention group and 600 patients in the control group. Sample sizes varied from 46 to 194 individuals across the studies. Six studies involved samples of over 100 breast cancer/head and neck cancer patients.

Three studies involved interventions with heparin moisturizers, 4 involved aloe vera gel, 1 involved a new multi-active moisturizer with hyaluronic acid as the main moisturizing ingredient, 2 involved hyaluronic acid lotion, 1 with a water-in-oil emulsion, and 2 used barrier-repair moisturizers. Table 1 presents detailed basic characteristics of the included studies.

**Table 1:** Main characteristics of the included studies.

First author	Year	Study design	Location	Cancer type	Intervention		Cases		Radiotherapy dose	Treatment duration	Outcome	Measures of effect
					Experimental group	Control group	Experimental group	Control group				
Deantonio, L.^[38]^	2025	RCT	Switzerland	Breast Cancer	Hyaluronic acid 0.2 % cream	Standard skin care (neutral comparator cream)	43	43	50 Gy, 2 Gy/fraction, 5 days per week for 5 consecutive wk or 40 Gy, 2.667 Gy/fraction, 5 d per wk for 3 consecutive wk	5 wk	1. The severity of ARD at the final RT session 2. Skin-related QOL	1.ARD regraded by RTOG. 2.Patients’ QoL was measured by the 36-Item Short Form Health Survey (SF-36).
Qian Li^[26]^	2024	RCT	China	Breast Cancer	Barrier-Repair Moisturizer (Episil ®)	Standard skin care	65	35	50–60Gy, in a fractionated manner with a single dose of 2Gy	5–6 wk	1. Severity of ARD (Incidence and grading of ARD during treatment) 2. Skin-related QOL 3. Degree of itchiness 4. Skin pain	1.ARD regraded by RTOG. 2.QOL was measured using the European Organization for Research and Treatment of Cancer Quality of Life Scale (EORTC QLQ-C30, version 3). 3.Itchiness and skin pain were assessed using the numerical rating scale assessment tool.
Robijns^[27]^	2023	RCT	Belgium	Breast Cancer	Novel, multi-active emollient	Standard skin care (hydroactive colloid gel Flamigel®)	50	50	16 × 2.66 Gy + 5 × 2.66 Gy	NR	1. The severity of ARD at the final RT session 2. Skin-related QOL 3. Patient symptoms (degree of itchiness)	1.ARD regraded by RTOG. 2.Patients’ QOL was evaluated using the Skindex-29 questionnaire. 3.The researchers evaluated the severity of pruritus based on the NCI-CTCAE v5.
Tsai^[28]^	2023	RCT	China	Head and Neck Cancer	Barrier-Repair Moisturizer (Comfeel® Barrier Cream)	No treatment	26	23	The average prescribed radiation dose was 6218 cGy (ranging from 5000–7000 cGy)	NR	Severity of ARD	ARD regraded by RISRAS and RTOG.
Kawamori^[29]^	2021	RCT	Japan	Breast Cancer	Heparinoid moisturizer	No treatment	35	37	42.56 Gy in 16 fractions	3 wk	1. Severity of ARD 2. Skin-related QOL (quality of life)	Dermatology Life Quality Index (DLQI; range 0–30, Higher scores indicate worsening quality of daily life).
Rahimi^[30]^	2020	RCT	USA	Breast Cancer	Novel hyaluronan cream	Standard skin care (placebo cream)	28	28	50.4 Gy in 28 fractions + 10 Gy in 5 fractions	5 wk	Severity of ARD	NCI-CTCAE v.4.0 scale.
Mohamed^[31]^	2020	RCT	Egypt	Breast Cancer	Aloe vera gel	Standard skin care (receiving routine hospital care)	66	66	20 × 2 Gy	4 wk	Severity of ARD	ARD regraded by RISRAS.
Ogita^[32]^	2019	RCT	Japan	Breast Cancer	Heparinoid moisturizer	No treatment	14	32	48–50 Gy in 24–25 fractions	5 wk	Sebum content and sebum composition	1.Sebumeter® is used for measuring sebum content. 2.Sebum composition and content were analyzed by chromatography (HP1100 Agilent Technologies) and evaporative light scattering detector (ELSD, SofTA 300SM&S Instruments Inc.).
Sekiguchi^[33]^	2018	RCT	Japan	Breast Cancer	Heparinoid moisturizer	No treatment	14	32	2 Gy 5 d a wk up to 48–50 Gy with photons	5 wk	Skin moisture levels	Corneometer CM825®(Courage + Khazaka, Cologne, Germany).
Ahmadloo^[34]^	2017	RCT	Iran	Breast Cancer	Aloe vera gel	No treatment	50	50	A total dose of 50 Gy with a daily fraction of 2 Gy, with 5 fractions per wk	5 wk	Severity of ARD	Acute Radiation Morbidity Scoring Criteria.
Hoopfer^[35]^	2015	RCT	Canada	Breast Cancer	Aloe vera gel	Standard skin care (placebo cream.)	81	77	45–50 Gy in 20–25 fractions	4–5 wk	Severity of ARD	Modified 10-point Catterall skin scoring profile.
Jensen^[36]^	2011	RCT	Germany	Breast Cancer	Oil-in-Water Emulsion (WO1932)	No treatment	34	30	NR	NR	Skin moisture levels	Corneometer® and the Tewameter® TM210 (both Courage and Khazaka, Cologne, Germany).
Williams^[37]^	1996	RCT	USA	Breast Cancer	Aloe vera gel	Standard skin care (aplacebo gel.)	97	97	Planned dose including 4 doses (45–50 Gy, 50.01–56 Gy, 56.01–60 Gy, > 60 Gy), dose fraction including 1.8–2.0 Gy and <1.8 cGy)	NR	Severity of ARD	ARD regraded by RTOG.

ARD = acute radiation dermatitis, NCI-CTCAE V5 = National Cancer Institute Common Terminology Criteria for Adverse Events Version 5, NR = not reported, NRS = numerical rating scale, QOL = quality of life, RCT = randomized controlled trials, RISRAS = radiation induced skin reaction assessment scale, RTOG = radiation therapy oncology group.

### 3.3. Quality assessment

**Figure 2.**
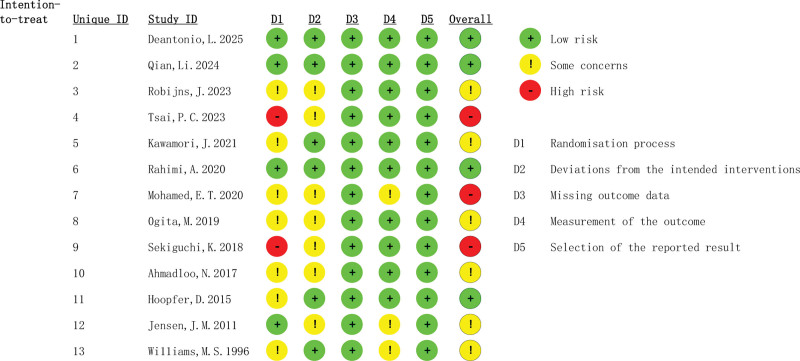
Risk of bias summary review author’s judgements about each risk of bias item for each included study.

**Figure 3.**
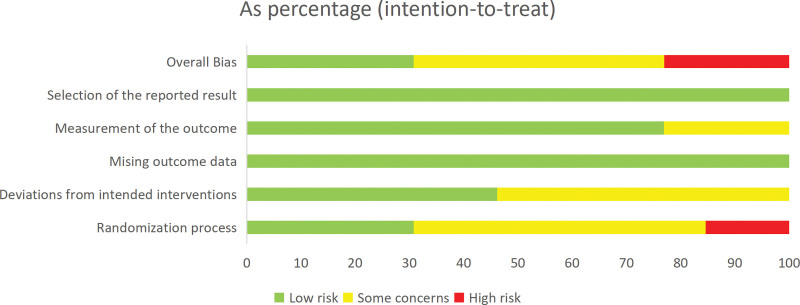
General chart of methodological quality assessment.

The Cochrane Risk of Bias Assessment Tool, RoB2.0, was used to evaluate the risk of bias in the final 12 included studies. Software generated bias risk ratio plots (Fig. 2) and methodological quality assessment summary plots (Fig. 3). All 13 studies included were RCTs. Among them, 4^[26,30,35,38]^ studies were of high quality. They had a low risk of bias in random sequence generation, blinding, allocation concealment, completeness of outcome data, and selective reporting of study results. Six^[27,29,32,34,36,37]^ studies had an unclear risk of bias overall due to unclear procedures for participant selection based on random sequence or unclear data completeness. Two^[28,33]^ studies were rated as having a high risk of bias due to lack of allocation concealment, and 1^[31]^ study with more than 3 domains of “some concerns” were also rated as having a high risk. Selective reporting of trial results was rated as low risk for all studies, and completeness of outcome data was rated as low risk for all studies, despite some loss to follow-up.

### 3.4. Meta-analysis results of included studies

#### 3.4.1. Meta-analysis results of acute radiodermatitis incidence and grading

According to the Radiation Therapy Oncology Group (RTOG) criteria. Studies using alternative grading systems (e.g., RISRAS, NCI-CTCAE) were reclassified into RTOG grades to ensure consistency. ARD Grade 0: No dermatitis, Grade 1 to 2: Mild to moderate dermatitis (faint erythema, dry desquamation, or tender/vivid erythema), Grade ≥ 3: Severe dermatitis (moist desquamation, ulceration, or necrosis).

#### 3.4.2. ARD Grade 0

**Figure 4.**
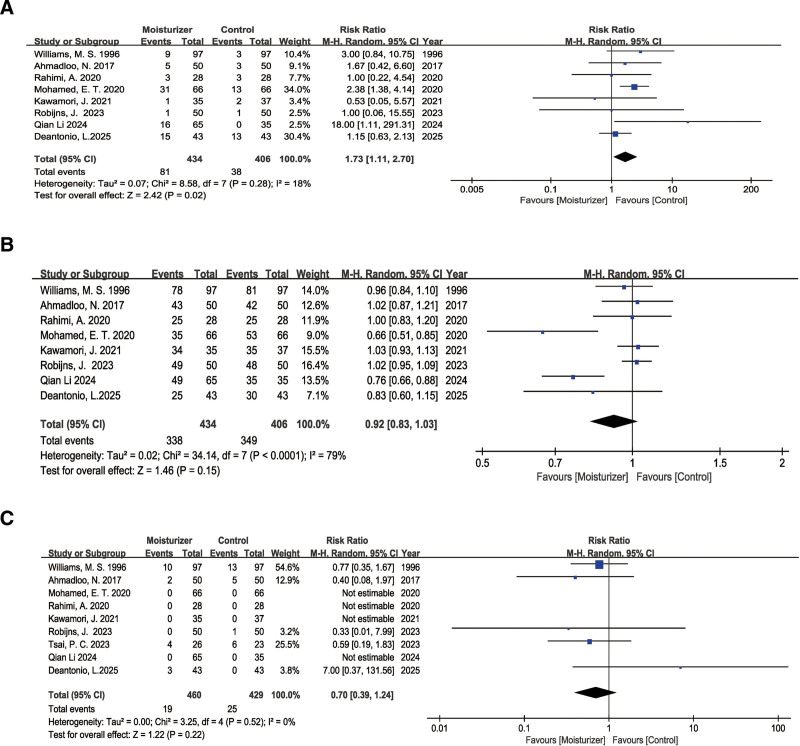
Forest plot of the use of moisturizer in ARD. (A) Forest plot of the use of moisturizer in grade 0 ARD. The vertical lines indicate the pooled summary estimate (95% Cl) for all studies in (A) (95% CI 1.11–2.70; *P* = .02). The area of each square is proportional to the inverse variance of the estimate. The horizontal lines indicate the 95% CIs of the estimate; (B) Forest plot of the use of moisturizer in grade 1 to 2 ARD. The vertical lines indicate the pooled summary estimate (95% CI) for all studies in (B) (95% CI,0.83–1.03; *P* = .15). The area of each square is proportional to the inverse variance of the estimate. The horizontal lines indicate the 95% CIs of the estimate; (C) Forest plot of the use of moisturizer in grade ≥3 ARD. The vertical lines indicate the pooled summary estimate (95% Cl) for all studies in (C) (95% CI 0.39–1.24; *P* = .22). The area of each square is proportional to the inverse variance of the estimate. The horizontal lines indicate the 95% CIs of the estimate. ARD = acute radiation dermatitis, CI = confidence interval.

We included 8 studies,^[26,27,29–31,34,37,38]^ all RCTs, with a combined total of 840 study subjects, comprising 434 in the experimental group and 406 in the control group. we used a random-effect model for all outcomes. Meta-analysis of the incidence of grade 0 radiodermatitis (i.e., no dermatitis) indicated a significantly higher probability of grade 0 radiodermatitis occurrence in the moisturizer group compared to the control group (RR = 1.73, 95% CI [1.11–2.70], *P* = .02) (Fig. 4A).

#### 3.4.3. ARD Grades 1 to 2

Eight studies were included,^[26,27,29–31,34,37,38]^ all RCTs, with a combined total of 840 study subjects, comprising 434 in the experimental group and 406 in the control group. Meta-analysis of the incidence of grades 1 to 2 radiodermatitis (mild to moderate dermatitis) showed that compared to the control group, there was a lower probability of grades 1 to 2 radiodermatitis occurrence in the moisturizer group. However, the difference was not statistically significant (RR = 0.92, 95% CI [0.83–1.03], *P* = .15) (Fig. 4B).

#### 3.4.4. ARD Grade ≥3

Nine studies were included,^[26–31,34,37,38]^ all RCTs, with a combined total of 889 study subjects, comprising 460 in the experimental group and 429 in the control group. Meta-analysis of the grade ≥ 3 radiodermatitis incidence indicated that compared to the control group. However, there was a lower probability of grade ≥ 3 radiodermatitis occurrence in the moisturizer group; the difference was not statistically significant (RR = 0.70, 95% CI [0.39–1.24], *P* = .22) (Fig. 4C).

#### 3.4.5. Subgroup analysis of ARD

Since the moisturizers in this study involved various types, including hyaluronic acid-based moisturizers, heparin sodium-based moisturizers, natural aloe vera gel moisturizers, and Episil skin barrier moisturizers, all of which have moisturizing effects but may differ in their active ingredients, we further conducted a subgroup analysis. Based on the primary substances responsible for their moisturizing effects, we categorized them into barrier-repair moisturizers, high molecular weight moisturizers, and natural soothing moisturizers to explore potential differences.

Subgroup analysis indicated that both barrier-repair and natural soothing moisturizers were effective in preventing the occurrence of ARD (RR = 18.0, 95% CI [1.11–291.31], *P* = .04; RR = 2.36, 95% CI [1.47–3.79], *P* = .0004), while no significant difference was observed between the moisturizer group and the control group for high molecular weight moisturizers (RR = 1.08, 95% CI [0.63–1.86], *P* = .78) (Fig. S1, Supplemental Digital Content, https://links.lww.com/MD/R403).

As for the incidence of grade 1 to 2 ARD, barrier-repair moisturizers were the only group that effectively reduced the occurrence of grade 1 to 2 ARD (RR = 0.76, 95% CI [0.66–0.88], *P* = .0002), while no significant effect was observed for the other 2 moisturizer groups on the incidence of grade 1 to 2 ARD (RR = 1.01, 95% CI [0.95–1.08], *P* = .69; RR = 0.89, 95% CI [0.71–1.11], *P* = .29) (Fig. S2, Supplemental Digital Content, https://links.lww.com/MD/R403).

Similarly, in the subgroup analysis of the incidence of grade ≥ 3 ARD, none of the moisturizers in the 3 subgroups showed a positive effect on the occurrence of grade ≥ 3 ARD compared to the control group (RR = 0.59, 95% CI [0.19–1.83], *P* = .36; RR = 1.63, 95% CI [0.08, 32.52, *P* = .75]; RR = 0.68, 95% CI [0.34–1.36], *P* = .28) (Fig. S3, Supplemental Digital Content, https://links.lww.com/MD/R403).

### 3.5. Meta-analysis results of itching severity

**Figure 5.**
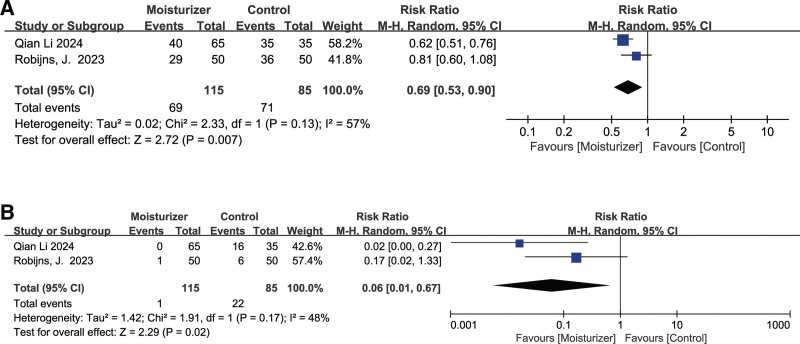
Forest plot of the use of moisturizer in itchiness grade. (A) Forest plot of the use of moisturizer in itchiness; (B) Forest plot of the use of moisturizer in itchiness ≥2 grade.

Pruritus severity: Graded using study-specific scales (NCI-CTCAE), with severe pruritus defined as ≥ Grade 2. We included 2 studies^[26,27]^ both RCTs, with a combined total of 200 study subjects, comprising 115 in the experimental group and 85 in the control group. Meta-analysis of the overall incidence of itching showed that compared to the control group, the moisturizer group had a lower probability of itching occurrence, with a statistically significant difference (RR = 0.69, 95% CI [0.53–0.90], *P* = .007) (Fig. 5A). Specifically, the moisturizer group had a lower probability of experiencing severe itching (≥2 grade) compared to the control group, with a statistically significant difference (RR = 0.06, 95% CI [0.01–0.67], *P* = .02) (Fig. 5B). However, there was no statistically significant difference in the occurrence of no itching (grade 0) (RR = 5.32, 95% CI [0.13–211.1], *P* = .37), or moderate itching (grade 1) (RR = 1.02, 95% CI [0.80–1.31], *P* = .85) between the 2 groups.

### 3.6. Meta-analysis results of quality of life

**Figure 6.**
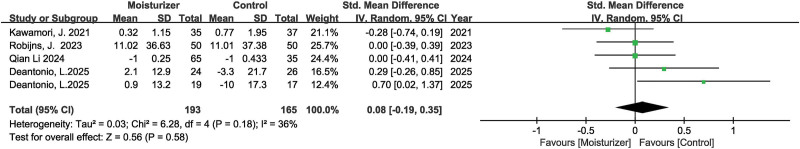
Forest plot of the use of moisturizer in QoL. QoL = quality of life.

Skin-related QoL: Assessed using validated instruments such as the Dermatology Life Quality Index (DLQI) or EORTC QLQ-C30. We included 4 studies,^[26,27,29,38]^ all RCTs, with a combined total of 358 study subjects, comprising 193 in the experimental group and 165 in the control group. Meta-analysis of the QOL showed that there was no statistically significant difference in the QOL between the moisturizer group and the control group (SMD = 0.08, 95% CI [−0.19 to 0.35], *P* = .58) (Fig. 6).

### 3.7. Meta-analysis results of skin water content

Measured via devices such as the Corneometer®. Two studies were included,^[33,36]^ both RCTs, with a combined total of 110 study subjects, comprising 48 in the experimental group and 62 in the control group. Meta-analysis of skin water content indicated that there was no statistically significant difference in skin water content between the moisturizer group and the control group (MD = −0.42, 95% CI [−5.53 to 4.69], *P* = .16) (Fig. S4, Supplemental Digital Content, https://links.lww.com/MD/R403).

### 3.8. Sebum content

Ogita et al conducted a randomized controlled trial,^[32]^ including 46 study subjects, with 14 in the experimental group and 32 in the control group. They evaluated the efficacy of heparinoid moisturizer treatment for radiation dermatitis by measuring sebum content in the stratum corneum using a sebum detector after whole breast radiotherapy (WBRT). At baseline, the mean sebum content of the irradiated breasts in both groups was similar (experimental group: 9.6 ± 10.6 μg/cm^2^, control group: 12.3 ± 16.5 μg/cm^2^). After 4 weeks of radiotherapy, the sebum content in the experimental group increased to 15.2 ± 15.1 μg/cm^2^, while it continued to decrease in the control group to 0.4 ± 0.7 μg/cm^2^, with a statistically significant difference (*P* <.001).

### 3.9. Publication bias and sensitivity analysis

Since the number of included studies was <10, funnel plot and Egger test were not performed. We searched ClinicalTrials.gov, WHO ICTRP, and ChiCTR, and found no completed but unpublished relevant trials. For the included studies, we compared their published study protocols/registration information, and found no evidence of selective reporting of main outcomes. Therefore, there is currently no obvious indication of publication bias, but due to the limited number of studies, it cannot be completely ruled out. We conducted sensitivity analyses by systematically removing each included study 1 at a time and reevaluating the pooled effect estimates. The consistency of effect estimates across all sensitivity analyses supports the robustness of our primary findings. And the complete sensitivity analysis results demonstrating the consistency of effect estimates across all iterations are provided in Supplementary Material 2, Supplemental Digital Content, https://links.lww.com/MD/R400.

### 3.10. Rating the body of evidence

The evidence for the incidence of ARD was rated moderate to very low. The evidence was downgraded for serious risk of bias (all grades ARD), serious inconsistency (grade1–2), and serious inprecision (grade1–2 and ≥ 3). For itching severity the evidence was rated low. The evidence was downgraded for serious inconsistency and serious inprecision. The evidence was rated moderate for QOL. The quality of evidence was downgraded for serious inprecision. And the evidence for water content was rated very low, because of serious risk of bias, serious inconsistency, and serious inprecision (Table 2).

**Table 2:** Quality of evidence for meta-analysis.

Outcomes	No. of studies	Limitations	Inconsistency	Indirectness	Inprecision	Publication bias	Effect size (95% CI)	No. of participants	Quality of the evidence (GRADE)
ARD Grade 0	8	Serious limitations (1 high risk of bias, 4 some concers)	No serious inconsistency	No serious indirectness	No serious inprecision	Undetected	RR = 1.73, 95% CI (1.11–2.70)	840 (Experimental group: 434; Control group:406)	⊕⊕⊕○ Moderate
ARD Grades 1–2	8	Serious limitations (1 high risk of bias, 4 some concers)	Serious inconsistency*	No serious indirectness	Serious inprecision†	Undetected	RR = 0.92, 95% CI (0.83–1.03)	840 (Experimental group: 434; Control group:406)	⊕○○○ Very low
ARD Grade ≥ 3	9	Serious limitations (2 high risk of bias, 4 some concers)	No serious inconsistency	No serious indirectness	Serious inprecision†	Undetected	RR = 0.70, 95% CI (0.39, 1.24)	889 (Experimental group: 460; Control group:429)	⊕⊕○○ Low
Itching Severity	2	No serious limitation	Serious inconsistency*	No serious indirectness	Serious inprecision‡	Undetected	RR = 0.69, 95% CI (0.53, 0.90)	200 (Experimental group: 115; Control group:85)	⊕⊕○○ Low
Itching Severity ≥ 2	2	No serious limitation	Serious inconsistency*	No serious indirectness	Serious inprecision‡	Undetected	RR = 0.06, 95% CI (0.01, 0.67)	200 (Experimental group: 115; Control group:85)	⊕⊕○○ Low
Quality of Life	4	No serious limitation	No serious inconsistency	No serious indirectness	Serious inprecision†	Undetected	SMD = 0.08, 95% CI (−0.19 to 0.35)	358 (Experimental group: 193; Control group:165)	⊕⊕⊕○ Moderate
Water Content	2	Serious limitations (1 high risk of bias, 1 some concers)	Serious inconsistency*	No serious indirectness	Serious inprecision‡	Undetected	MD = −0.77, 95% CI (−4.52 to 2.99)	110 (Experimental group: 48; Control group:62)	⊕○○○ Very low

* Serious inconsistency when heterogeneity *I*^2^ was >40%.

† 95% CI overlap with a small effect size of 0.2 for beneficial and detrimental effects.

‡ Serious imprecision for <400 participants.

## 4. Discussion

Radiation dermatitis occurs due to exposure to beta, gamma, and X-rays, resulting in an inflammatory reaction of the skin and mucous membranes.^[39,40]^

Moisturizers function primarily by reducing transepidermal water loss. Upon application to compromised skin, the lipid components in moisturizers create a protective layer on the skin surface, This process not only supports the regeneration of the skin barrier but also effectively relieves dryness and shields the skin from both internal and external irritants.^[41]^ High-quality moisturizers can play a crucial role as adjunct treatments in both the management and prevention of such disorders, while also mitigating symptoms like pruritus and stinging sensations.^[42]^

We conducted a comprehensive review of existing literature. Previous meta-analyses^[33]^ have explored the impact of moisturizers on radiodermatitis, but there are few RCTs, the sample sizes are relatively small, and the outcome measures discussed are limited. In contrast, our study expanded the sample size and our findings confirmed the positive impact of moisturizers in ARD treatment. Specifically, compared to the control group, the use of moisturizers demonstrated a certain degree of improvement in both the severity level of ARD and itching symptoms. However, no statistically significant differences were observed between the moisturizer and control groups in other indicators, such as QOL and skin water content.

Sekiguchi et al study^[33]^ suggested that although there was a decrease in the incidence rates of ARD at grades ≥ 2 and ≥ 3 in the moisturizer group compared to the control group, the difference between the 2 groups was not statistically significant. However, we believed that their conclusion was not rigorous, and the interpretation of the data was inaccurate. The results of our meta-analysis showed that the moisturizer group had a statistically significant higher probability of not developing ARD compared with the control group. This suggested that clinicians might consider incorporating moisturizers when they devise treatment and care plans for patients.

However, no significant statistical difference was observed between the moisturizer group and the control group for grades 1 to 2 and ≥3 ARD, aligning with the findings of Sekiguchi et al study. Furthermore, we identified high heterogeneity among the groups for grades 1 to 2 ARD, potentially attributable to several factors: varied RT techniques, scheduling, and patterns may impact RD risk.^[43]^ Modern radiation techniques, such as intensity-modulated radiation therapy, ensure dose uniformity within the irradiated field, while adopting hyperfractionation schemes has reduced ARD severity.^[44,45]^ In our review, the ≥ grade 3 ARD incidence rate in the moisturizer group was 4.1%, compared to 5.8% in the control group. Given the inherently low probability of severe radiation dermatitis occurrence, this led to the lack of significant statistical difference in ≥ grade 3 ARD occurrence between the moisturizer and control groups. Dermatitis severity assessment was somewhat indirect. Among the 9 studies, 4 studies adopted RTOG while 5 used other criteria, including RISRAS^[46]^ and NCI-CTCAE. We reclassified them according to the RTOG system, potentially contributing to study heterogeneity.

Our subgroup analysis indicates that high molecular weight moisturizers (such as those based on hyaluronic acid or heparin) do not significantly prevent ARD, in stark contrast to the effectiveness observed in barrier-repair and natural soothing moisturizers. This may be attributed to the molecular size of these compounds, which could hinder deep skin penetration and effective moisturization when the skin barrier is compromised by radiation.^[21]^ Furthermore, the studies involving these moisturizers had smaller sample sizes, which may have led to a lack of statistical power to detect significant effects. Contrary to our findings, previous literature has indicated varying effects of high molecular weight moisturizers on skin hydration and barrier function, highlighting the need for further investigation through more rigorously designed trials and larger patient cohorts.^[27,32]^ Clinically, these results suggest a need to reconsider moisturizer recommendations for patients undergoing radiotherapy, prioritizing formulations proven to be more effective in preventing ARD. The analysis of pruritus symptoms in this meta-analysis review reveals that compared to the control group, the moisturizing group exhibits a lower overall probability of experiencing itching and a reduced likelihood of severe itching (≥2 grades).^[30]^ This suggests that moisturizing measures are somewhat effective in alleviating pruritus symptoms. When the skin suffers damage from ionizing radiation, significant changes occur in the function and structure of its cells and tissues. These alterations lead to the activation of nerve fibers surrounding the dermal-epidermal junction. Subsequent damage to skin cells and ensuing inflammatory responses stimulate nerve endings, resulting in the sensation of itching.^[47,48]^ Adequately moisturized skin experiences improved barrier function, significantly reducing sensitivity to external stimuli and consequently alleviating itching symptoms.^[49]^

We also analyzed the patients’ QOL. The randomized controlled trial by Qian Li et al^[26]^ demonstrated that the moisturizing group exhibited a reduction in rates of stress, anxiety, and depression, significantly improving the QOL of patients after radiotherapy. In assessing patient QOL, we utilized 2 standardized questionnaires, namely the DLQI^[50]^ and Skindex-19,^[51]^ while validated, may lack the sensitivity to detect subtle, radiotherapy-specific changes in symptoms and daily comfort that are relevant over a limited treatment period. Second, the widespread adoption of modern, conformal radiotherapy techniques has likely reduced the baseline severity of dermatitis in contemporary study populations; when symptoms are predominantly mild, the measurable signal on QOL scales may be inherently limited. Finally, the total sample size across studies remains relatively small, resulting in limited statistical power to detect a modest but clinically meaningful effect.

Adequate hydration is crucial for maintaining skin health. In normal skin, its ability to retain moisture primarily relies on the outermost layer of the epidermis, the stratum corneum, which acts as a barrier to prevent water loss.^[52]^ However, ionizing radiation directly damages epidermal and follicular cells (especially stem cells), skin appendages, fibroblasts, and endothelial cells, severely compromising the skin’s barrier function in retaining moisture.^[53]^ The primary function of moisturizers is to improve the hydration of the intercellular matrix. In determining the stratum corneum’s water content, the meta-analysis in this review revealed no significant statistical difference between the moisturizing group and the control group. This lack of significant difference may be attributed to several factors. Firstly, the limited number of included studies comprising only 2 research papers involving 110 study subjects. Secondly, the timing of follow-up assessments varied considerably among the included studies. For example, 1 study^[33]^ evaluated stratum corneum hydration on the first day and at 2 weeks postradiation, whereas another study^[36]^ conducted assessments on days 1, 8, and 47 following irradiation. Moreover, the natural rehydration of the stratum corneum tends to progress over time after radiation exposure, and this recovery trajectory is significantly influenced by individual patient variability.

While this study complied with recognized standards for systematic reviews and meta-analyses, some limitations may still exist. Despite expanding the sample size included in this review, the sample size for specific key outcome measures, such as QOL and skin water content, is still relatively small, which may lead to insufficient representativeness. Robijns, J. et al noted that when patients experienced severe inflammatory skin itching, corticosteroid ointments were prescribed to 5 out of 50 control group patients and 3 out of 50 experimental group patients. Using glucocorticoid medications may interfere with assessing other outcome measures, such as ARD grading and QOL. According to the study by Tsai et al (31), there were differences in self-care abilities and adherence to moisturizing skincare between male and female head and neck cancer patients. Female patients generally exhibit more potent self-care abilities and a higher willingness to use moisturizers. However, considering that only one of the 12 studies we included involved head and neck cancer patients, while the rest were all female breast cancer patients, this impact is overall minimal. The studies we included exhibit diversity in terms of types of moisturizers, tumor types, radiotherapy doses, intervention implementation methods, and blinding. Although all the RCTs are considered to have relatively reasonable quality, using different measurement tools in various studies may lead to differences in reported outcomes. Additionally, some studies lacked original data, and although we attempted to obtain and verify this data by contacting the authors, this issue still poses a certain level of impact. Notably, our study included a vehicle-controlled trial which was necessary because the vehicle can influence drug efficacy, risk of irritation, hydration potential, suitability for different body parts, and patient preferences.^[54]^ Future research will benefit from more randomized, double-blind, and vehicle-controlled trials, including standard care control groups matched to the treatment group vehicle, to reduce bias and enhance the credibility of the evidence.

It is difficult to completely rule out the risk of publication bias in this study. On 1 hand, traditional funnel plots and Egger test are not applicable due to the insufficient number of included studies (<10); on the other hand, although we searched clinical trial registry platforms and study protocols, there was no direct evidence of unpublished trials or selective reporting of results, but the sensitivity of these methods is limited. Therefore, the possibility of publication bias still needs to be treated with caution.

The prevention and treatment of radiation dermatitis remain challenging. Current studies on various topical medications and wound dressings yield inconsistent results, making it challenging to establish comprehensive, evidence-based practice guidelines.^[55]^ Most current interventions and recommendations are based on clinical experience rather than solid data support.^[56]^ Regardless of the severity of the condition, basic management strategies should be implemented for every patient at risk of RD. These strategies include cleansing the skin, using moisturizers to maintain skin water content, and avoiding contact with irritants.^[57]^ The United States Cutaneous Oncology Management algorithm recommends that topical products used for managing ARD should possess moisturizing properties and be free of allergens and fragrances.^[58,59]^ All moisturizers restore water content through 4 basic mechanisms: occlusion, humectancy, hydrophilic matrix, and photoprotection.^[60]^ High-quality moisturizers should be applied gently and be capable of retaining moisture over prolonged periods while aiding in the recovery of normal skin barrier function.^[61]^ In recent years, increasing attention has been given to the role of lipid components – such as ceramides – and the lamellar structures between keratinocytes in maintaining barrier integrity and skin homeostasis.^[62]^ Consequently, an optimal moisturizer should integrate the moisturizing properties of natural moisturizing factors, the occlusive effects of lipid membranes, and the structural support of intercellular lipids, offering a multifaceted approach to protecting and repairing compromised skin.^[63,64]^

We can provide skin care instructions for patients undergoing radiotherapy through written or electronic brochures, treatment diaries, and smartphone applications. This approach not only emphasizes the importance of moisturizers but also guides patients on how to use them properly. Moreover, it offers symptomatic treatment advice for patients experiencing irritation, thus reducing inconsistencies in information dissemination among healthcare professionals.^[65]^

Future research should focus on thoroughly exploring the effects of moisturizers on the physiological and physical parameters of the skin while providing conclusive evidence for selecting topical moisturizers, medications, and dressings for preventing and treating ARD. Incorporating current insights into the underlying pathophysiology of ARD, future studies should also evaluate the effects of interventions on skin barrier function and the skin microbiome, as well as comprehensively evaluate outcomes based on objective reports from both clinicians and patients.

## 5. Conclusion

ARD is one of the most common side effects experienced by patients undergoing RT treatment. Despite biases and confounding factors present in the analyzed 12 clinical trials, research suggests that using moisturizers during RT appears safe and beneficial, albeit with little certainty of evidence. Future meticulous RCTs should be implemented to obtain higher quality evidence, employing sensitive assessment tools to generate high-quality evidence. This study provides essential guidance for preventing and treating RD while offering valuable insights for subsequent research.

## Acknowledgments

We would like to thank James Foster, who works at BoSiHan International Education Ltd., for his help proofreading this paper.

## Author contributions

**Conceptualization:** Wangli Wu.

**Data curation:** Xiuwen Zhang.

**Formal analysis:** Lin Yao.

**Investigation:** Saisai Liu.

**Methodology:** Xiaojing Sun.

**Project administration:** Xuehong Yu.

**Resources:** Xiuwen Zhang.

**Software:** Lin Yao.

**Supervision:** Xuehong Yu.

**Writing – original draft:** Wangli Wu.

**Writing – review & editing:** Wangli Wu, Xuehong Yu.

## Supplementary Material
