# Supplementary material for: The effect of moisturizers on acute radiation dermatitis: A systematic review and meta-analysis
Source: Medicine (Baltimore). 2026 Feb 20;105(8):e47688. doi: 10.1097/MD.0000000000047688 (PMC12928883; doi:10.1097/MD.0000000000047688)

**Appendix 1**

**Search strategy for Pubmed**

| **No** | **Query** | **Results** |
| --- | --- | --- |
| #1 | ((("Skin Cream"[Mesh]) OR (Skin Cream[Title/Abstract])) OR (Skin Lotion[Title/Abstract])) OR (Dermal Cream[Title/Abstract]) | 1782 |
| #2 | ((((((((("Gels"[Mesh]) OR (Gels[Title/Abstract])) OR (gel[Title/Abstract])) OR (gel matrix[Title/Abstract])) OR (gelcosponge[Title/Abstract])) OR (haven gel[Title/Abstract])) OR (hydraulic gel[Title/Abstract])) OR (hydrocarbon gel[Title/Abstract])) OR (hydron gel[Title/Abstract])) OR (oxygel[Title/Abstract]) | 405054 |
| #3 | (((((("Emollients"[Mesh]) OR (Emollients[Title/Abstract])) OR (emollient[Title/Abstract])) OR (emollient agent[Title/Abstract])) OR (moisturizer[Title/Abstract])) OR (moisturizing cream[Title/Abstract])) OR (emollient cream[Title/Abstract]) | 4817 |
| #4 | ((((((((((((((((((((((((((((((((((((((((((((((((((((((((((((((((((((((((((((((("Hyaluronic Acid"[Mesh]) OR (Hyaluronic Acid[Title/Abstract])) OR (Amo Vitrax[Title/Abstract])) OR (Biolon[Title/Abstract])) OR (Etamucine[Title/Abstract])) OR (Hyaluronan[Title/Abstract])) OR (Hyvisc[Title/Abstract])) OR (Luronit[Title/Abstract])) OR (Sodium Hyaluronate[Title/Abstract])) OR (Hyaluronate Sodium[Title/Abstract])) OR (Amvisc[Title/Abstract])) OR (Healon[Title/Abstract])) OR (adant[Title/Abstract])) OR (adant dispo[Title/Abstract])) OR (amo vitrax[Title/Abstract])) OR (arthrease[Title/Abstract])) OR (artz[Title/Abstract])) OR (bionect[Title/Abstract])) OR (clearvisc[Title/Abstract])) OR (duovisc[Title/Abstract])) OR (durolane[Title/Abstract])) OR (eyecon[Title/Abstract])) OR (go-on (drug[Title/Abstract]))) OR (halonix[Title/Abstract])) OR (healon gv[Title/Abstract])) OR (healon yellow[Title/Abstract])) OR (healon5[Title/Abstract])) OR (healonid[Title/Abstract])) OR (hialid[Title/Abstract])) OR (hyalcon[Title/Abstract])) OR (hyalein[Title/Abstract])) OR (hyalgal[Title/Abstract])) OR (hyalgan[Title/Abstract])) OR (hyalovet[Title/Abstract])) OR (hyalubrix[Title/Abstract])) OR (hyaluronate[Title/Abstract])) OR (hyaluronic acid component[Title/Abstract])) OR (hyladerm[Title/Abstract])) OR (hylaform[Title/Abstract])) OR (hylan g f 20[Title/Abstract])) OR (hylan g-f 20[Title/Abstract])) OR (hylartin v[Title/Abstract])) OR (hylo-comod[Title/Abstract])) OR (hylumed[Title/Abstract])) OR (hyruan[Title/Abstract])) OR (ialugen[Title/Abstract])) OR (juvederm[Title/Abstract])) OR (lagricel ofteno[Title/Abstract])) OR (laservis[Title/Abstract])) OR (me 3710[Title/Abstract])) OR (monovisc[Title/Abstract])) OR (na hylan[Title/Abstract])) OR (na-hylan[Title/Abstract])) OR (nrd 101[Title/Abstract])) OR (nrd101[Title/Abstract])) OR (ophthalin[Title/Abstract])) OR (ophthalin plus[Title/Abstract])) OR (orthovisc[Title/Abstract])) OR (ostenil[Title/Abstract])) OR (perlane[Title/Abstract])) OR (potassium hyaluronate[Title/Abstract])) OR (provisc[Title/Abstract])) OR (radiaplexrx[Title/Abstract])) OR (restylane[Title/Abstract])) OR (restylane lyft[Title/Abstract])) OR (si 4402[Title/Abstract])) OR (sinovial[Title/Abstract])) OR (sl 1010[Title/Abstract])) OR (sperm select[Title/Abstract])) OR (supartz[Title/Abstract])) OR (suplasyn[Title/Abstract])) OR (synocrom[Title/Abstract])) OR (synojoynt[Title/Abstract])) OR (synvisc[Title/Abstract])) OR (teosyal[Title/Abstract])) OR (triluron[Title/Abstract])) OR (unihylon[Title/Abstract])) OR (viscoseal[Title/Abstract])) OR (vismed[Title/Abstract])) OR (vitrax[Title/Abstract]) | 49651 |
| #5 | #1 OR #2 OR #3 OR #4 | 453514 |
| #6 | (((((((((((((((("Radiodermatitis"[Mesh]) OR (Radiodermatitis[Title/Abstract])) OR (Radiodermatitides[Title/Abstract])) OR (Radiation-Induced Dermatitis[Title/Abstract])) OR (Radiation Induced Dermatitis[Title/Abstract])) OR (Radiation-Induced Dermatitides[Title/Abstract])) OR (Radiation Recall Dermatitis[Title/Abstract])) OR (Radiation Recall Dermatitides[Title/Abstract])) OR (Radiation Recall Reaction[Title/Abstract])) OR (radio dermatitis[Title/Abstract])) OR (radiodermitis[Title/Abstract])) OR (roentgen dermatitis[Title/Abstract])) OR (roentgen dermatosis[Title/Abstract])) OR (x radiation dermatitis[Title/Abstract])) OR (x ray dermatitis[Title/Abstract])) OR (x ray dermatosis[Title/Abstract])) OR (radiation dermatitis[Title/Abstract]) | 3902 |
| #7 | #5 AND #6 | 179 |

**Search strategy for Cochrane**

| **No** | **Query** | **Results** |
| --- | --- | --- |
| #1 | MeSH descriptor: [Radiodermatitis] explode all trees | 313 |
| #2 | (Radiodermatitis):ti,ab,kw OR (Radiodermatitides):ti,ab,kw OR (Radiation-Induced Dermatitis):ti,ab,kw OR (Radiation Induced Dermatitis):ti,ab,kw OR (Radiation-Induced Dermatitides):ti,ab,kw OR (Radiation Recall Dermatitis):ti,ab,kw OR (Radiation Recall Dermatitides):ti,ab,kw OR (Radiation Recall Reaction):ti,ab,kw OR (radio dermatitis):ti,ab,kw OR (radiodermitis):ti,ab,kw OR (roentgen dermatitis):ti,ab,kw OR (roentgen dermatosis):ti,ab,kw OR (x radiation dermatitis):ti,ab,kw OR (x ray dermatitis):ti,ab,kw OR (x ray dermatosis):ti,ab,kw OR (radiation dermatitis):ti,ab,kw | 1308 |
| #3 | #1 OR #2 | 1308 |
| #4 | MeSH descriptor: [Skin Cream] explode all trees | 493 |
| #5 | (Skin Cream):ti,ab,kw OR (Skin Lotion):ti,ab,kw OR (Dermal Cream):ti,ab,kw | 5788 |
| #6 | MeSH descriptor: [Gels] explode all trees | 3337 |
| #7 | (Gels):ti,ab,kw OR (gel):ti,ab,kw OR (gel matrix):ti,ab,kw OR (gelcosponge):ti,ab,kw OR (haven gel):ti,ab,kw OR (hydraulic gel):ti,ab,kw OR (hydrocarbon gel):ti,ab,kw OR (hydron gel):ti,ab,kw OR (oxygel):ti,ab,kw | 19782 |
| #8 | MeSH descriptor: [Emollients] explode all trees | 662 |
| #9 | (Emollients):ti,ab,kw OR (emollient):ti,ab,kw OR (emollient agent):ti,ab,kw OR (moisturizer):ti,ab,kw OR (moisturizing cream):ti,ab,kw OR (emollient cream):ti,ab,kw | 2277 |
| #10 | MeSH descriptor: [Hyaluronic Acid] explode all trees | 2450 |
| #11 | (Hyaluronic Acid):ti,ab,kw OR (Amo Vitrax):ti,ab,kw OR (Biolon):ti,ab,kw OR (Etamucine):ti,ab,kw OR (Hyaluronan):ti,ab,kw OR (Hyvisc):ti,ab,kw OR (Luronit):ti,ab,kw OR (Sodium Hyaluronate):ti,ab,kw OR (Hyaluronate Sodium):ti,ab,kw OR (Amvisc):ti,ab,kw OR (Healon):ti,ab,kw OR (adant):ti,ab,kw OR (adant dispo):ti,ab,kw OR (amo vitrax):ti,ab,kw OR (arthrease):ti,ab,kw OR (artz):ti,ab,kw OR (bionect):ti,ab,kw OR (clearvisc):ti,ab,kw OR (duovisc):ti,ab,kw OR (durolane):ti,ab,kw OR (eyecon):ti,ab,kw OR (halonix):ti,ab,kw OR (healon gv):ti,ab,kw OR (healon yellow):ti,ab,kw OR (healon5):ti,ab,kw OR (healonid):ti,ab,kw OR (hialid):ti,ab,kw OR (hyalcon):ti,ab,kw OR (hyalein):ti,ab,kw OR (hyalgal):ti,ab,kw OR (hyalgan):ti,ab,kw OR (hyalovet):ti,ab,kw OR (hyalubrix):ti,ab,kw OR (hyaluronate):ti,ab,kw OR (hyaluronic acid component):ti,ab,kw OR (hyladerm):ti,ab,kw OR (hylaform):ti,ab,kw OR (hylan g f 20):ti,ab,kw OR (hylan g-f 20):ti,ab,kw OR (hylartin v):ti,ab,kw OR (hylo-comod):ti,ab,kw OR (hylumed):ti,ab,kw OR (hyruan):ti,ab,kw OR (ialugen):ti,ab,kw OR (juvederm):ti,ab,kw OR (lagricel ofteno):ti,ab,kw OR (laservis):ti,ab,kw OR (me 3710):ti,ab,kw OR (monovisc):ti,ab,kw OR (na hylan):ti,ab,kw OR (na-hylan):ti,ab,kw OR (nrd 101):ti,ab,kw OR (nrd101):ti,ab,kw OR (ophthalin):ti,ab,kw OR (ophthalin plus):ti,ab,kw OR (orthovisc):ti,ab,kw OR (ostenil):ti,ab,kw OR (perlane):ti,ab,kw OR (potassium hyaluronate):ti,ab,kw OR (provisc):ti,ab,kw OR (radiaplexrx):ti,ab,kw OR (restylane):ti,ab,kw OR (restylane lyft):ti,ab,kw OR (si 4402):ti,ab,kw OR (sinovial):ti,ab,kw OR (sl 1010):ti,ab,kw OR (sperm select):ti,ab,kw OR (supartz):ti,ab,kw OR (suplasyn):ti,ab,kw OR (synocrom):ti,ab,kw OR (synojoynt):ti,ab,kw OR (synvisc):ti,ab,kw OR (teosyal):ti,ab,kw OR (triluron):ti,ab,kw OR (unihylon):ti,ab,kw OR (viscoseal):ti,ab,kw OR (vismed):ti,ab,kw OR (vitrax):ti,ab,kw OR (go-on (drug)):ti,ab,kw | 6229 |
| #12 | #4 OR #5 OR #6 OR #7 OR #8 OR #9 OR #10 OR #11 | 32060 |
| #13 | #3 AND #12 | 321 |

**Search strategy for Embase**

| **No** | **Query** | **Results** |
| --- | --- | --- |
| #1 | 'radiation dermatitis'/exp | 6327 |
| #2 | 'radiodermatitis'/exp OR radiodermatitis OR radiodermatitides:ab,ti OR 'radiation-induced dermatitis':ab,ti OR 'radiation induced dermatitis':ab,ti OR 'radiation-induced dermatitides':ab,ti OR 'radiation recall dermatitis':ab,ti OR 'radiation recall dermatitides':ab,ti OR 'radiation recall reaction':ab,ti OR 'radio dermatitis':ab,ti OR radiodermitis:ab,ti OR 'roentgen dermatitis':ab,ti OR 'roentgen dermatosis':ab,ti OR 'x radiation dermatitis':ab,ti OR 'x ray dermatitis':ab,ti OR 'x ray dermatosis':ab,ti OR 'radiation dermatitis':ab,ti | 6929 |
| #3 | #1 OR #2 | 6929 |
| #4 | 'skin cream'/exp | 1105 |
| #5 | 'skin cream'/exp OR 'skin cream' OR (('skin'/exp OR skin) AND ('cream'/exp OR cream)) OR 'skin lotion':ab,ti OR 'dermal cream':ab,ti | 20329 |
| #6 | #4 OR #5 | 20329 |
| #7 | 'gel'/exp | 139595 |
| #8 | 'gels'/exp OR gels OR gel:ab,ti OR 'gel matrix':ab,ti OR gelcosponge:ab,ti OR 'haven gel':ab,ti OR 'hydraulic gel':ab,ti OR 'hydrocarbon gel':ab,ti OR 'hydron gel':ab,ti OR oxygel:ab,ti | 497144 |
| #9 | #7 OR #8 | 497144 |
| #10 | 'emollient agent'/exp | 8655 |
| #11 | 'emollients'/exp OR emollients OR emollient:ab,ti OR 'emollient agent':ab,ti OR moisturizer:ab,ti OR 'moisturizing cream':ab,ti OR 'emollient cream':ab,ti | 11622 |
| #12 | #10 OR #11 | 11622 |
| #13 | 'hyaluronic acid'/exp | 64932 |
| #14 | 'hyaluronic acid'/exp OR 'hyaluronic acid' OR (hyaluronic AND ('acid'/exp OR acid)) OR biolon:ab,ti OR etamucine:ab,ti OR hyaluronan:ab,ti OR hyvisc:ab,ti OR luronit:ab,ti OR 'sodium hyaluronate':ab,ti OR 'hyaluronate sodium':ab,ti OR amvisc:ab,ti OR healon:ab,ti OR adant:ab,ti OR 'adant dispo':ab,ti OR 'amo vitrax':ab,ti OR arthrease:ab,ti OR artz:ab,ti OR bionect:ab,ti OR clearvisc:ab,ti OR duovisc:ab,ti OR durolane:ab,ti OR eyecon:ab,ti OR ('go on':ab,ti AND drug:ab,ti) OR halonix:ab,ti OR 'healon gv':ab,ti OR 'healon yellow':ab,ti OR healon5:ab,ti OR healonid:ab,ti OR hialid:ab,ti OR hyalcon:ab,ti OR hyalein:ab,ti OR hyalgal:ab,ti OR hyalgan:ab,ti OR hyalovet:ab,ti OR hyalubrix:ab,ti OR hyaluronate:ab,ti OR 'hyaluronic acid component':ab,ti OR hyladerm:ab,ti OR hylaform:ab,ti OR 'hylan g f 20':ab,ti OR 'hylan g-f 20':ab,ti OR 'hylartin v':ab,ti OR 'hylo comod':ab,ti OR hylumed:ab,ti OR hyruan:ab,ti OR ialugen:ab,ti OR juvederm:ab,ti OR 'lagricel ofteno':ab,ti OR laservis:ab,ti OR 'me 3710':ab,ti OR monovisc:ab,ti OR 'na hylan':ab,ti OR 'nrd 101':ab,ti OR nrd101:ab,ti OR ophthalin:ab,ti OR 'ophthalin plus':ab,ti OR orthovisc:ab,ti OR ostenil:ab,ti OR perlane:ab,ti OR 'potassium hyaluronate':ab,ti OR provisc:ab,ti OR radiaplexrx:ab,ti OR restylane:ab,ti OR 'restylane lyft':ab,ti OR 'si 4402':ab,ti OR sinovial:ab,ti OR 'sl 1010':ab,ti OR 'sperm select':ab,ti OR supartz:ab,ti OR suplasyn:ab,ti OR synocrom:ab,ti OR synojoynt:ab,ti OR synvisc:ab,ti OR teosyal:ab,ti OR triluron:ab,ti OR unihylon:ab,ti OR viscoseal:ab,ti OR vismed:ab,ti OR vitrax:ab,ti | 76417 |
| #15 | #13 OR #14 | 76417 |
| #16 | #6 OR #9 OR #12 OR #15 | 588939 |
| #17 | #3 AND #16 | 551 |

**Search strategy for Web of Science**

| **No** | **Query** | **Results** |
| --- | --- | --- |
| #1 | TS=(Radiodermatitis) OR TS=(Radiodermatitides) OR TS=(Radiation-Induced Dermatitis) OR TS=(Radiation Induced Dermatitis) OR TS=(Radiation-Induced Dermatitides) OR TS=(Radiation Recall Dermatitis) OR TS=(Radiation Recall Dermatitides) OR TS=(Radiation Recall Reaction) OR TS=(radio dermatitis) OR TS=(radiodermitis) OR TS=(roentgen dermatitis) OR TS=(roentgen dermatosis) OR TS=(x radiation dermatitis) OR TS=(x ray dermatitis) OR TS=(x ray dermatosis) OR TS=(radiation dermatitis) | 4009 |
| #2 | TS=(Skin Cream) OR TS=(Skin Lotion) OR TS=(Dermal Cream) OR TS=(Gels) OR TS=(gel) OR TS=(gel matrix) OR TS=(gelcosponge) OR TS=(haven gel) OR TS=(hydraulic gel) OR TS=(hydrocarbon gel) OR TS=(hydron gel) OR TS=(oxygel) OR TS=(Emollients) OR TS=(emollient) OR TS=(emollient agent) OR TS=(moisturizer) OR TS=(moisturizing cream) OR TS=(emollient cream) | 577978 |
| #3 | TS=(Hyaluronic Acid) OR TS=(Amo Vitrax) OR TS=(Biolon) OR TS=(Etamucine) OR TS=(Hyaluronan) OR TS=(Hyvisc) OR TS=(Luronit) OR TS=(Sodium Hyaluronate) OR TS=(Hyaluronate Sodium) OR TS=(Amvisc) OR TS=(Healon) OR TS=(adant) OR TS=(adant dispo) OR TS=(amo vitrax) OR TS=(arthrease) OR TS=(artz) OR TS=(bionect) OR TS=(clearvisc) OR TS=(duovisc) OR TS=(durolane) OR TS=(eyecon) OR TS=(go-on (drug)) OR TS=(halonix) OR TS=(healon gv) OR TS=(healon yellow) OR TS=(healon5) OR TS=(healonid) OR TS=(hialid) OR TS=(hyalcon) OR TS=(hyalein) OR TS=(hyalgal) OR TS=(hyalgan) OR TS=(hyalovet) OR TS=(hyalubrix) OR TS=(hyaluronate) OR TS=(hyaluronic acid component) OR TS=(hyladerm) OR TS=(hylaform) OR TS=(hylan g f 20) OR TS=(hylan g-f 20) OR TS=(hylartin v) OR TS=(hylo-comod) OR TS=(hylumed) OR TS=(hyruan) OR TS=(ialugen) OR TS=(juvederm) OR TS=(lagricel ofteno) OR TS=(laservis) OR TS=(me 3710) OR TS=(monovisc) OR TS=(na hylan) OR TS=(na-hylan) OR TS=(nrd 101) OR TS=(nrd101) OR TS=(ophthalin) OR TS=(ophthalin plus) OR TS=(orthovisc) OR TS=(ostenil) OR TS=(perlane) OR TS=(potassium hyaluronate) OR TS=(provisc) OR TS=(radiaplexrx) OR TS=(restylane) OR TS=(restylane lyft) OR TS=(si 4402) OR TS=(sinovial) OR TS=(sl 1010) OR TS=(sperm select) OR TS=(supartz) OR TS=(suplasyn) OR TS=(synocrom) OR TS=(synojoynt) OR TS=(synvisc) OR TS=(teosyal) OR TS=(triluron) OR TS=(unihylon) OR TS=(viscoseal) OR TS=(vismed) OR TS=(vitrax) | 59652 |
| #4 | #3 OR #4 | 631620 |
| #5 | #2 AND #5 | 379 |

**Supplementary Figures**

Fig.S1 Subgroup analysis of grade 0 ARD

**
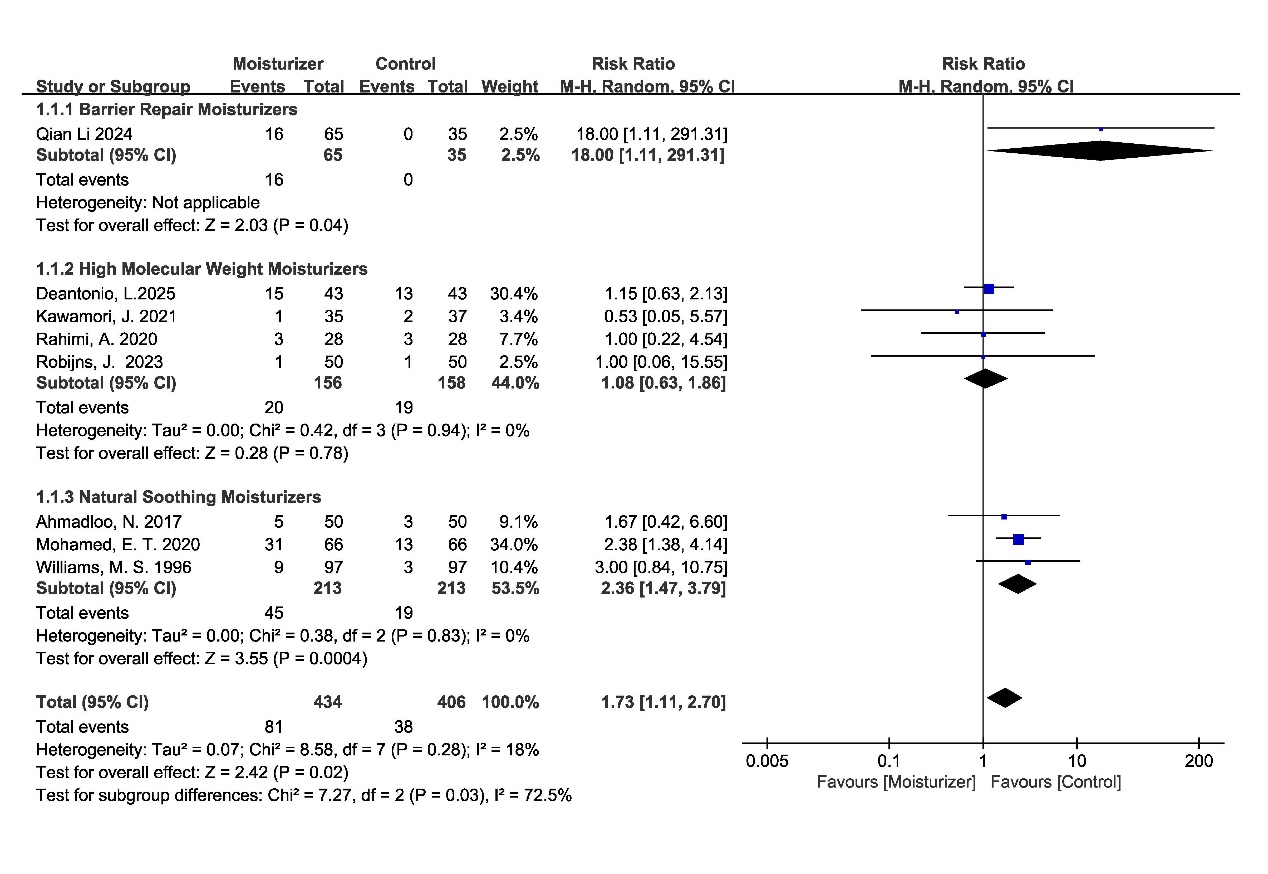
**

Fig.S2 Subgroup analysis of grade 1-2 ARD


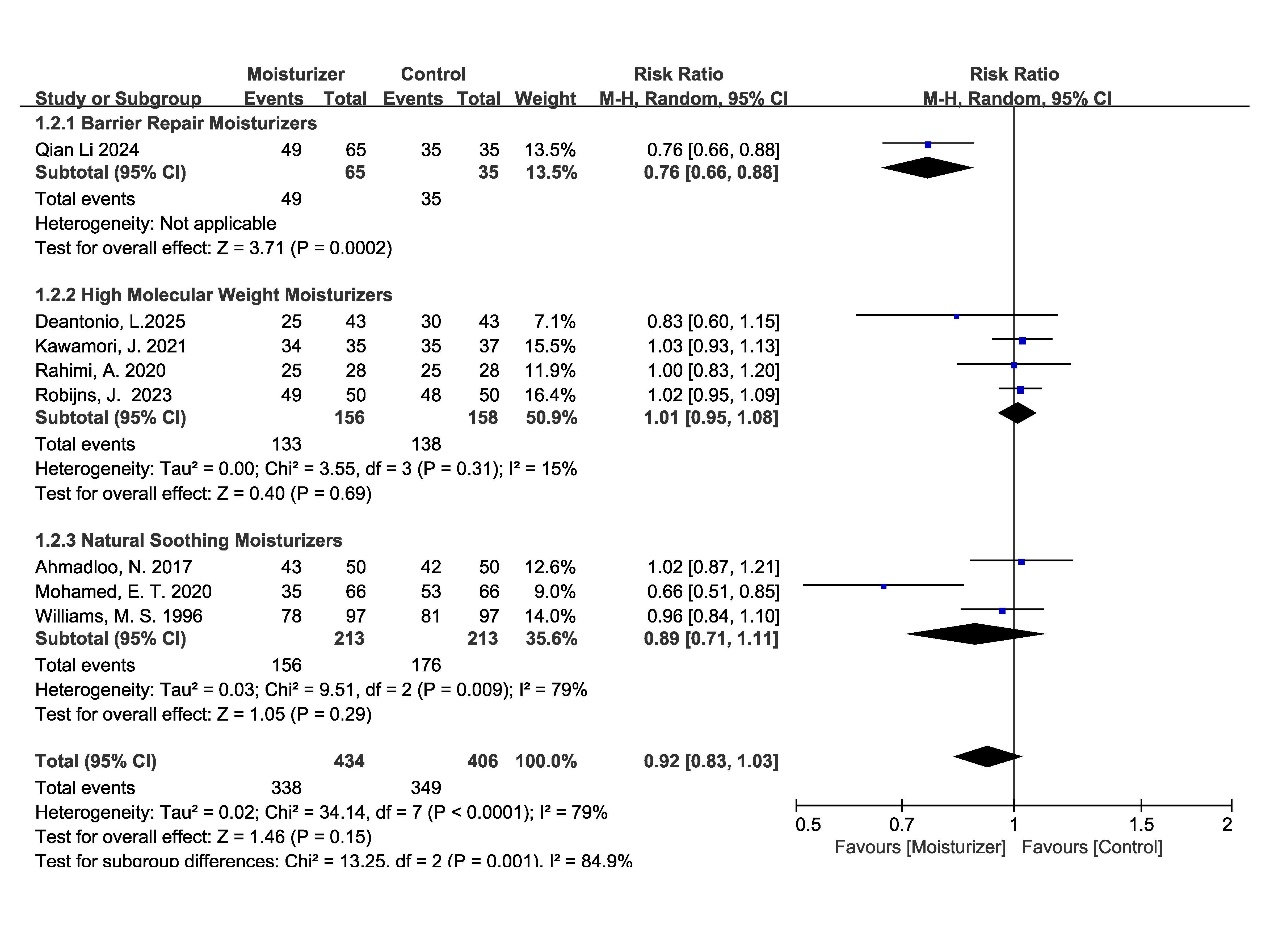


Fig.S3 Subgroup analysis of grade ≥3 ARD


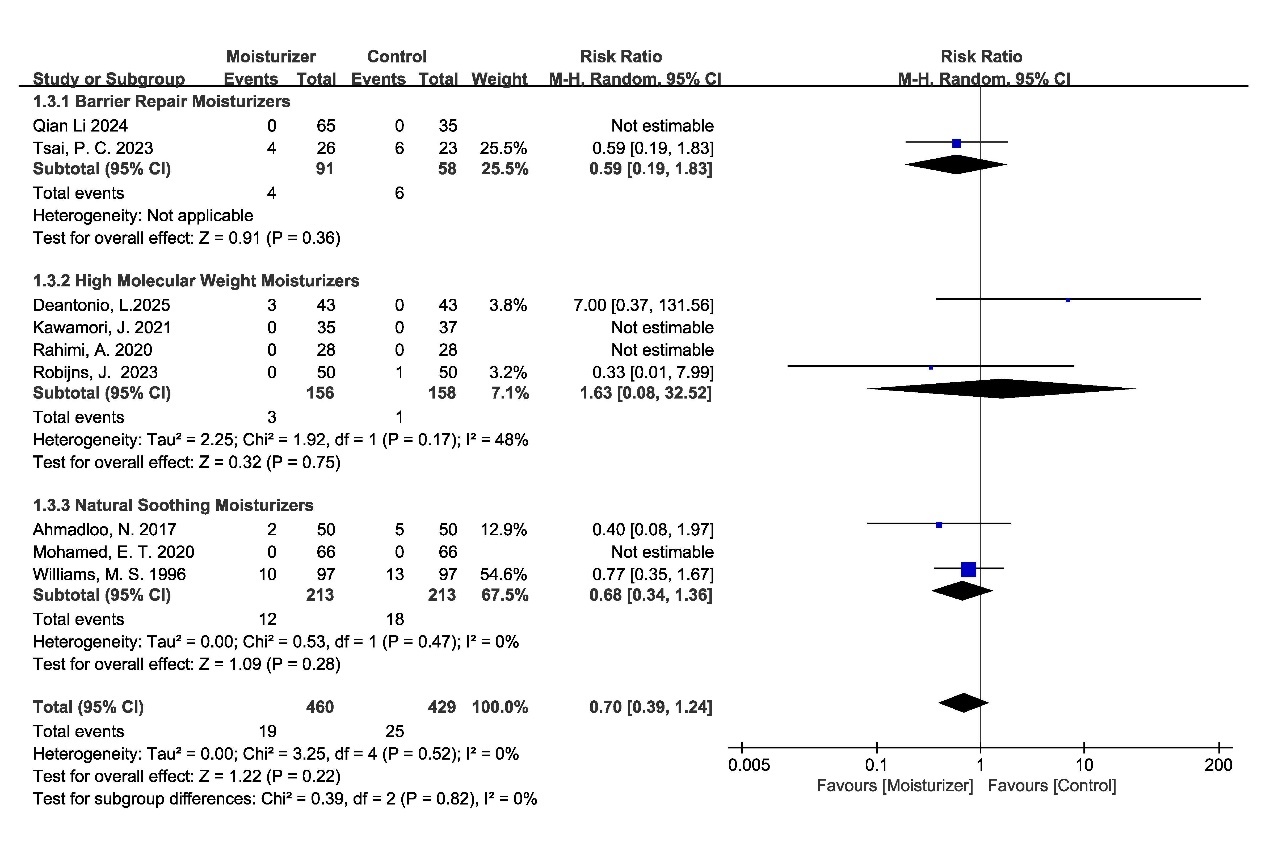


Fig.S4 Forest plot of the use of moisturizer in Skin Water Content


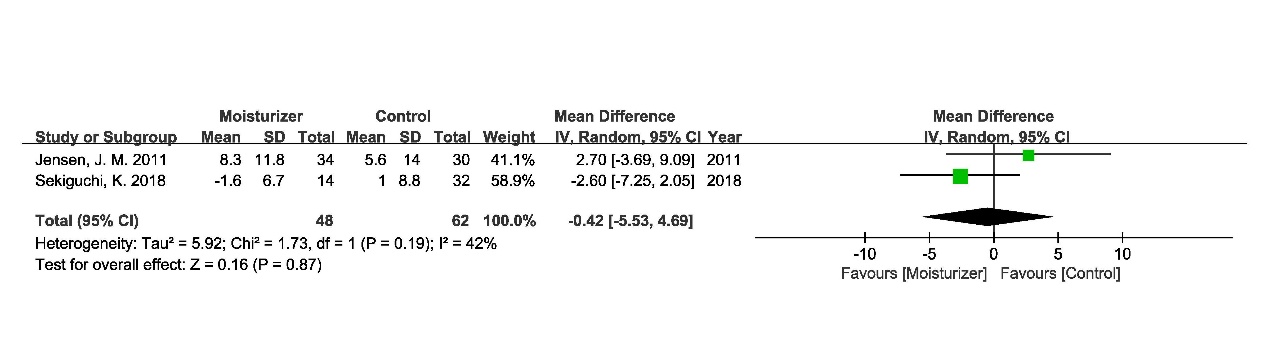

Supplement: Supplementary file 1 [file medi-105-e47688-s001.docx]
