# Supplementary material for: The effect of moisturizers on acute radiation dermatitis: A systematic review and meta-analysis
Source: Medicine (Baltimore). 2026 Feb 20;105(8):e47688. doi: 10.1097/MD.0000000000047688 (PMC12928883; doi:10.1097/MD.0000000000047688)
Supplement: Supplementary file 2 [file medi-105-e47688-s002.pdf]

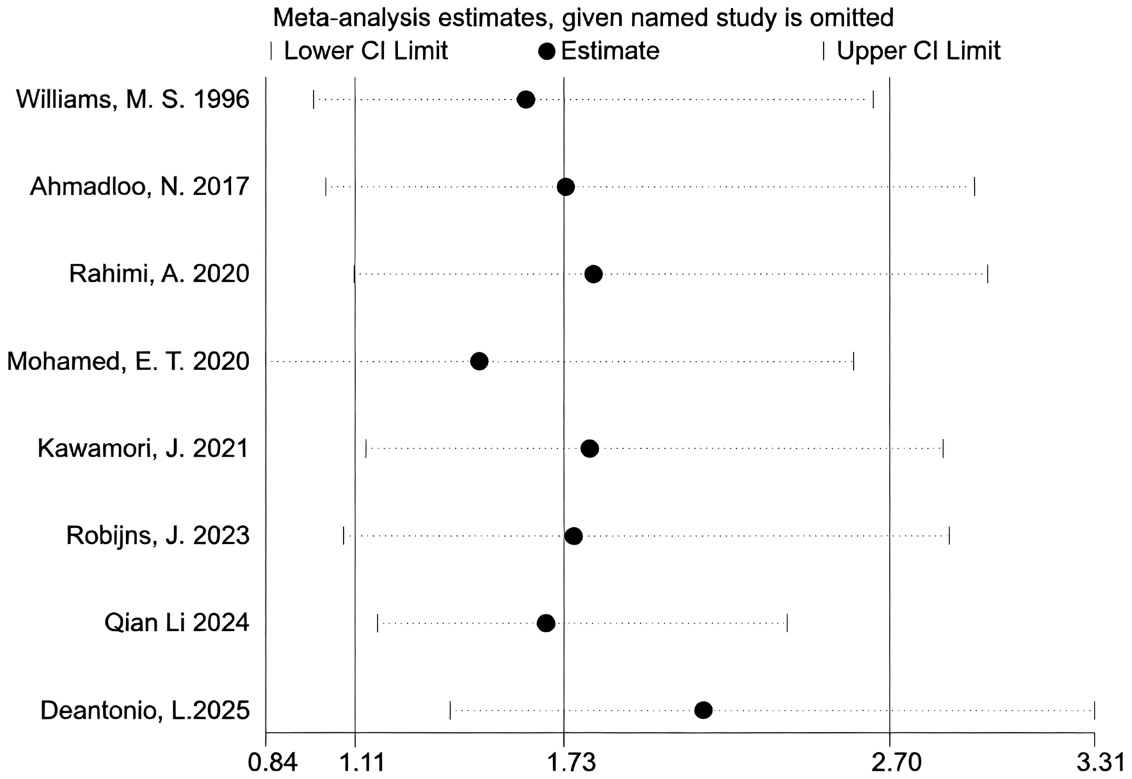

Fig S1 Sensitivity Analysis of Grade 0 ARD Incidence Rate

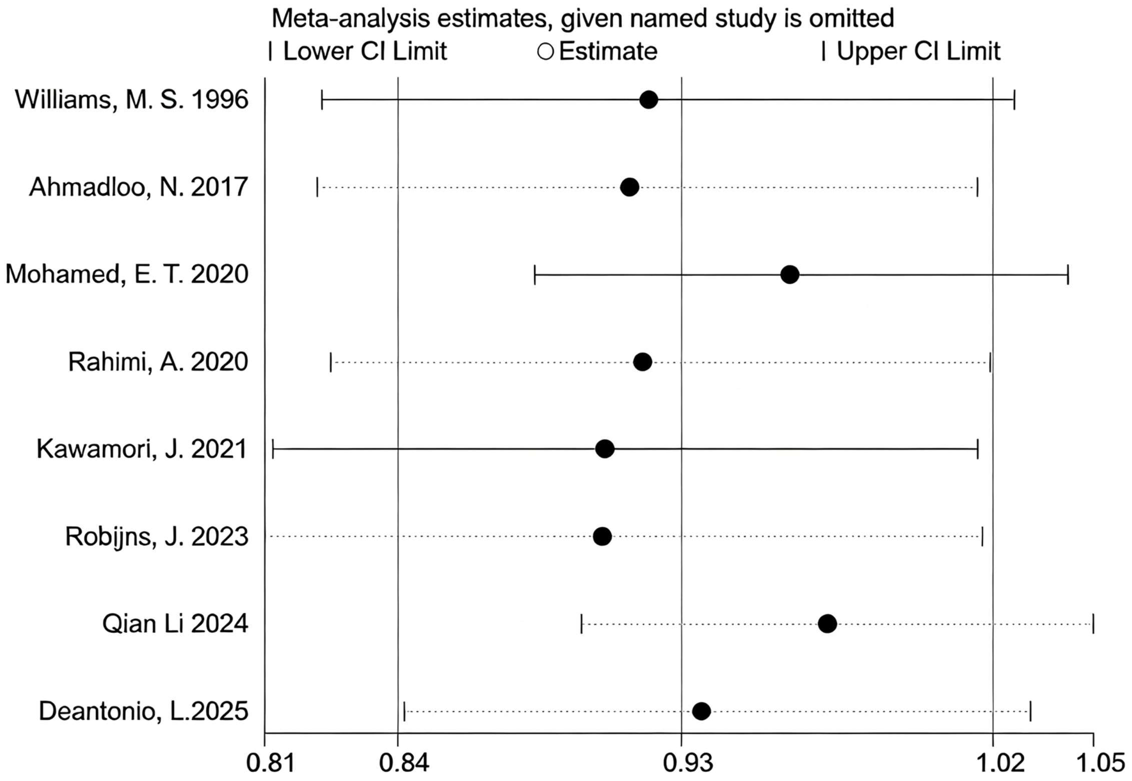

Fig S2 Sensitivity Analysis of Grade 1-2 ARD Incidence

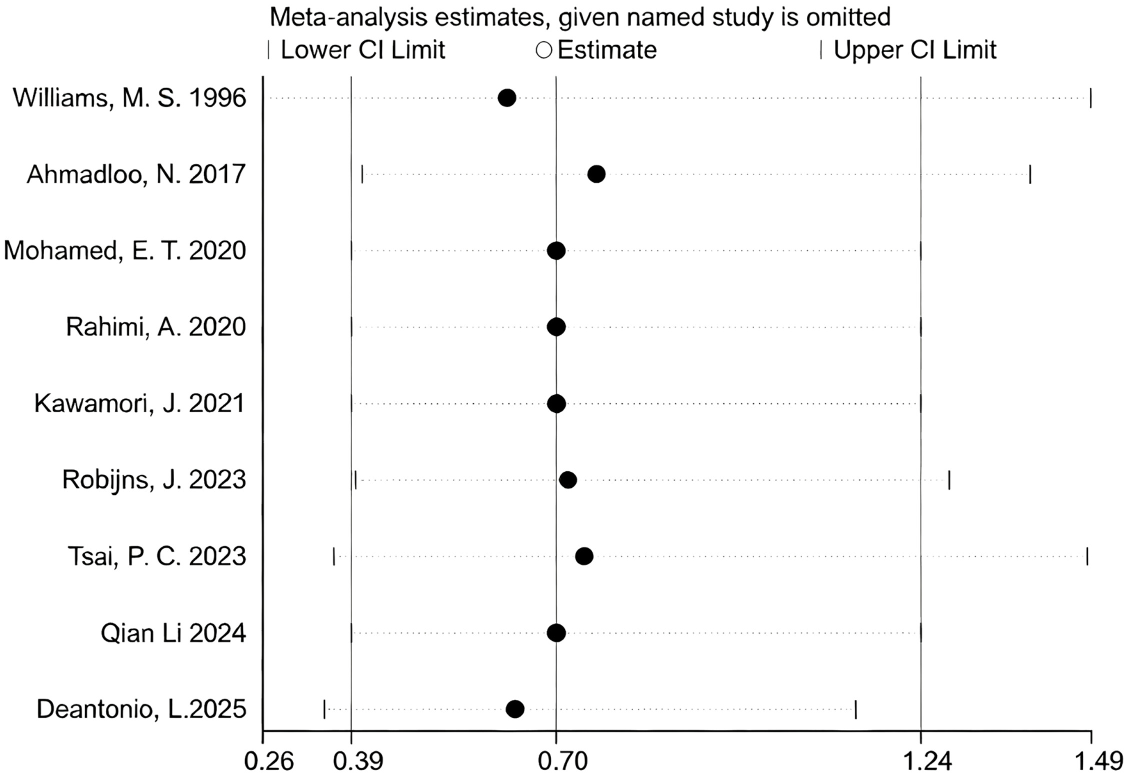

Fig S3 Sensitivity Analysis of Grade ≥3 ARD ARD Incidence Rate

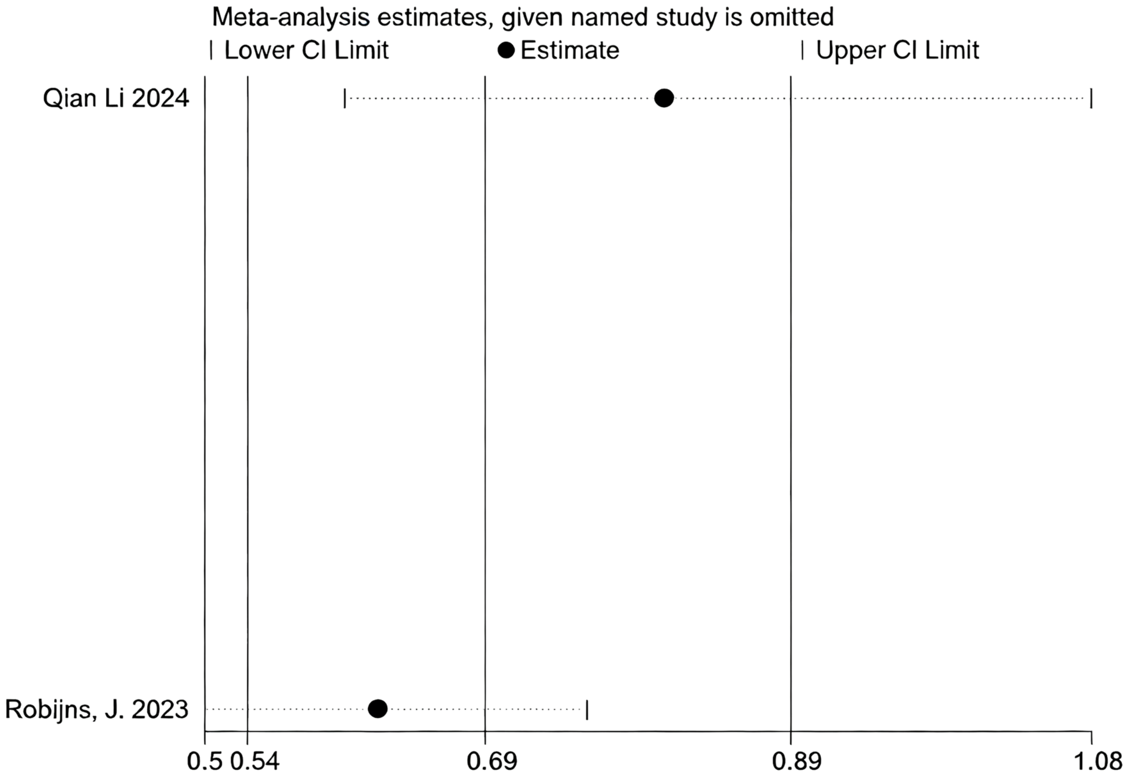

Fig S4 Sensitivity Analysis of itchiness Incidence Rate

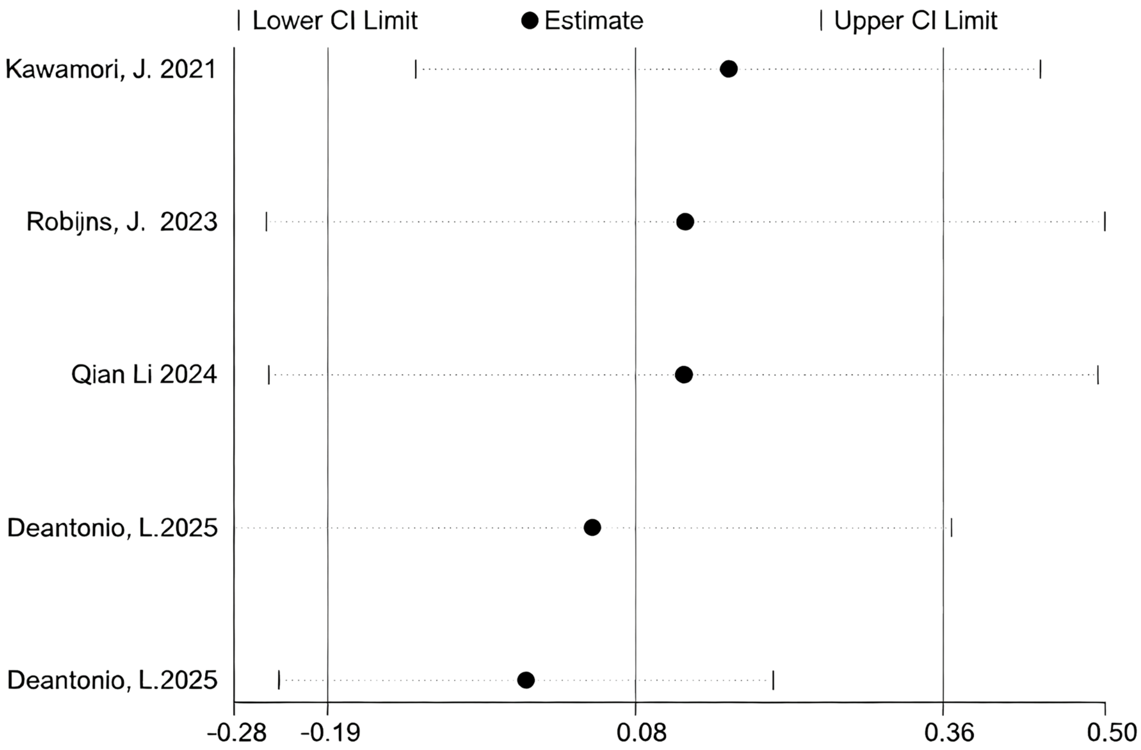

Fig S5 Sensitivity Analysis of QoL Incidence Rate

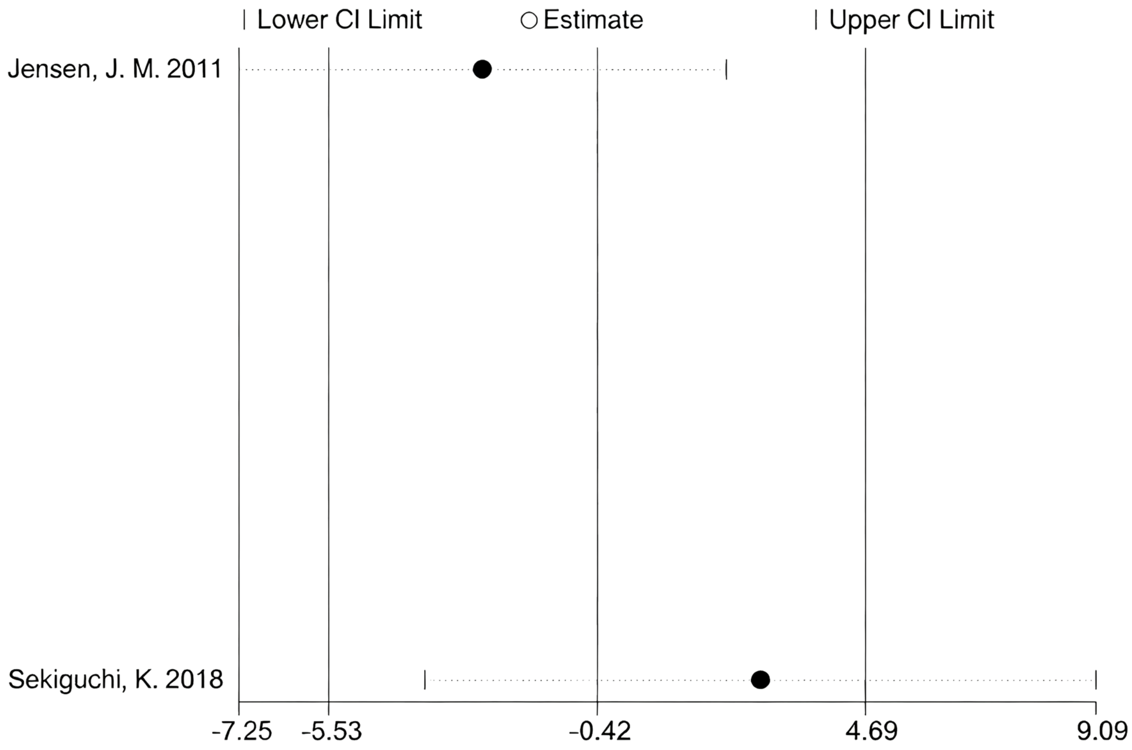

Fig S6 Sensitivity Analysis of Skin Water Content Incidence RatefO
